# Semimetal-triggered covalent interaction in Pt-based intermetallics for fuel-cell electrocatalysis

**DOI:** 10.1093/nsr/nwae233

**Published:** 2024-07-08

**Authors:** Han Cheng, Renjie Gui, Chen Chen, Si Liu, Xuemin Cao, Yifan Yin, Ruize Ma, Wenjie Wang, Tianpei Zhou, Xusheng Zheng, Wangsheng Chu, Yi Xie, Changzheng Wu

**Affiliations:** Key Laboratory of Precision and Intelligent Chemistry, School of Chemistry and Materials Science, University of Science and Technology of China, Hefei 230029, China; Key Laboratory of Precision and Intelligent Chemistry, School of Chemistry and Materials Science, University of Science and Technology of China, Hefei 230029, China; National Synchrotron Radiation Laboratory, University of Science and Technology of China, Hefei 230029, China; Chemistry Experiment Teaching Center, School of Chemistry and Materials Science, University of Science and Technology of China, Hefei 230029, China; Key Laboratory of Precision and Intelligent Chemistry, School of Chemistry and Materials Science, University of Science and Technology of China, Hefei 230029, China; Key Laboratory of Precision and Intelligent Chemistry, School of Chemistry and Materials Science, University of Science and Technology of China, Hefei 230029, China; Key Laboratory of Precision and Intelligent Chemistry, School of Chemistry and Materials Science, University of Science and Technology of China, Hefei 230029, China; National Synchrotron Radiation Laboratory, University of Science and Technology of China, Hefei 230029, China; Key Laboratory of Precision and Intelligent Chemistry, School of Chemistry and Materials Science, University of Science and Technology of China, Hefei 230029, China; National Synchrotron Radiation Laboratory, University of Science and Technology of China, Hefei 230029, China; National Synchrotron Radiation Laboratory, University of Science and Technology of China, Hefei 230029, China; Key Laboratory of Precision and Intelligent Chemistry, School of Chemistry and Materials Science, University of Science and Technology of China, Hefei 230029, China; Institute of Energy, Hefei Comprehensive National Science Center, Hefei 230026, China; Key Laboratory of Precision and Intelligent Chemistry, School of Chemistry and Materials Science, University of Science and Technology of China, Hefei 230029, China; Institute of Energy, Hefei Comprehensive National Science Center, Hefei 230026, China

**Keywords:** semimetals, Pt intermetallics, covalent interaction, oxygen reduction reaction, CO tolerance, fuel cells

## Abstract

Platinum-based intermetallic compounds (IMCs) play a vital role as electrocatalysts in a range of energy and environmental technologies, such as proton exchange membrane fuel cells. However, the synthesis of IMCs necessitates recombination of ordered Pt-M metallic bonds with high temperature driving, which is generally accompanied by side effects for catalysts’ structure and performance. In this work, we highlight that semimetal atoms can trigger covalent interactions to break the synthesis-temperature limitation of platinum-based intermetallic compounds and benefit fuel-cell electrocatalysis. Attributed to partial fillings of p-block in semimetal elements, the strong covalent interaction of d-p π backbonding can benefit the recombination of ordered Pt-M metallic bonds (PtGe, PtSb and PtTe) in the synthesis process. Moreover, this covalent interaction in metallic states can further promote both electron transport and orbital fillings of active sites in fuel cells. The semimetal-Pt IMCs were obtained with a temperature 300 K lower than that needed for the synthesis of metal-Pt intermetallic compounds and reached the highest CO-tolerant oxygen reduction activity (0.794 A mg^−1^ at 0.9 V and 5.1% decay under CO poisoning) among reported electrocatalysts. We anticipate that semimetal-Pt IMCs will offer new insights for the rational design of advanced electrocatalysts for fuel cells.

## Teaser

Semimetal atoms such as Ge, Sb and Te can trigger covalent interactions to break synthesis-temperature limitation of platinum-based intermetallic compounds and further bring CO tolerance for fuel-cell electrocatalysis.

## INTRODUCTION

Pt-based electrocatalysts play a vital role in a range of energy and environmental technologies, especially for proton exchange membrane fuel cells (PEMFCs) [1,2]. Great effort has been devoted to improving catalytic activity and decreasing the usage of Pt content [3,4], such as fabricating alloys with Fe, Co and Ni [5,6]. These Pt-M alloy catalysts have greatly improved the mass activity to a considerable degree compared with pure Pt [7–9]. However, Pt-M alloys with disordered atomic arrangement still suffer from poor stability, and the metal atoms easily etch in acid environments and further contribute to the collapse of the catalysts’ skeleton [10–12]. In this context, it is still highly urgent but challenging to synthesize Pt-based electrocatalysts with considerable activity and stability that can benefit the development of energy and environmental technologies [13–15].

In recent years, the Pt-based ordered structure has been regarded as having high intrinsic stability in fuel-cell electrocatalysis [16], in which the heteroatoms occupy specific sites covered by Pt atoms [17,18]. The synthesis principle for the Pt-based ordered structure can be divided by bonding heteroatoms. Non-metal elements, such as phosphorus [19] and selenium [20], are firstly used for synthesis of Pt-based covalent compounds. Nevertheless, the introduction of these non-metal elements with strong electronegativity is accompanied by electron localization effects [21], which suppress charge transport and further result in the decay of electrocatalytic activity [22]. On the other hand, transition metals are usually employed by fabricating intermetallic compounds with ordered structure for Pt [23–25]. Due to the metallic states of transition metals, delocalized electrons can travel between Pt-Metal metallic bonds, which benefits charge transport and further activity [26,27]. However, the formation of ordered metallic bonds between Pt and transition metals necessitates high temperature annealing (at more than 873 K), which generally leads to synthetic difficulties and structural side effects [28,29]. As a consequence, inhomogeneous particle size, order degree, element distribution and morphology can always be observed in Pt-based intermetallic compounds (IMCs), which cause Pt utilization efficiency and mass activity to decrease dramatically [30,31]. Therefore, breaking the limitation of synthesis temperature for Pt-based intermetallics can maximize electrocatalytic performance, making Pt-based intermetallics promising candidates for the next-generation of commercial catalysts.

**Figure 1.**
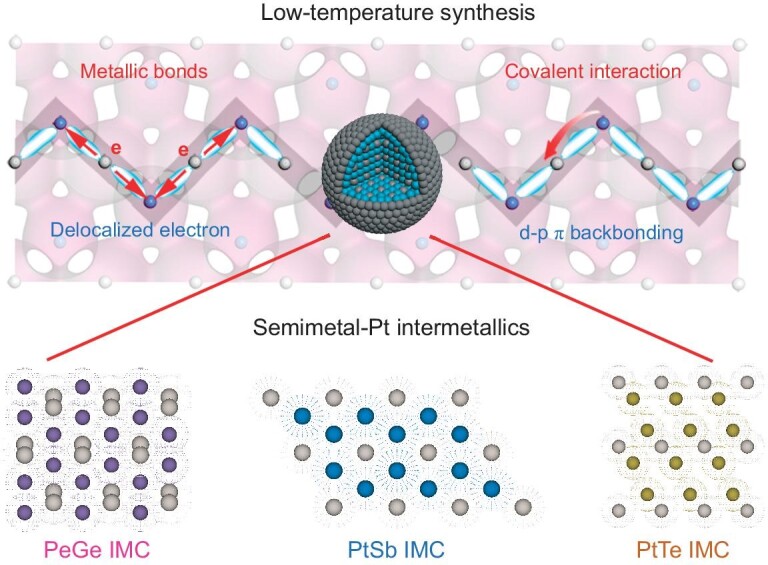
Scheme illustration. Covalent interaction and metallic states in semimetal-Pt intermetallic nanoparticles (PtGe, PtSb and PtTe IMCs) for low-temperature synthesis and fuel-cell electrolysis.

Semimetal elements, such as Ge, Sb and Te, combine the advantages of both metals and non-metals [32], with bonding strength between covalent bonds and metallic bonds [33]. In this work, we highlight the fact that the covalent interaction triggered by semimetals can break the temperature limitation in the synthesis of Pt-based intermetallic compounds and benefit fuel-cell electrocatalysis. A series of semimetal-Pt IMCs (PtGe, PtSb and PtTe nanoparticles) were obtained with a synthetic temperature 300 K lower (only half in Celsius) than that for metal-Pt intermetallic compounds, which is attributed to d-p π backbonding. This strong interaction can serve as a driving force for the recombination of ordered Pt-M metallic bonds. Moreover, benefiting from metallic states and covalent interactions in semimetal-Pt IMCs, electron transport and d-orbital fillings for Pt sites are regulated, which contributes to high performance in fuel-cell electrocatalysis (Fig. 1). We anticipate that the covalent interaction in semimetal-Pt IMCs could result in advanced electrocatalysts for practical fuel cells and offer new insights for the rational synthesis of intermetallic structure.

## RESULTS AND DISCUSSION

### Rational design of semimetal elements in Pt intermetallics

**Figure 2.**
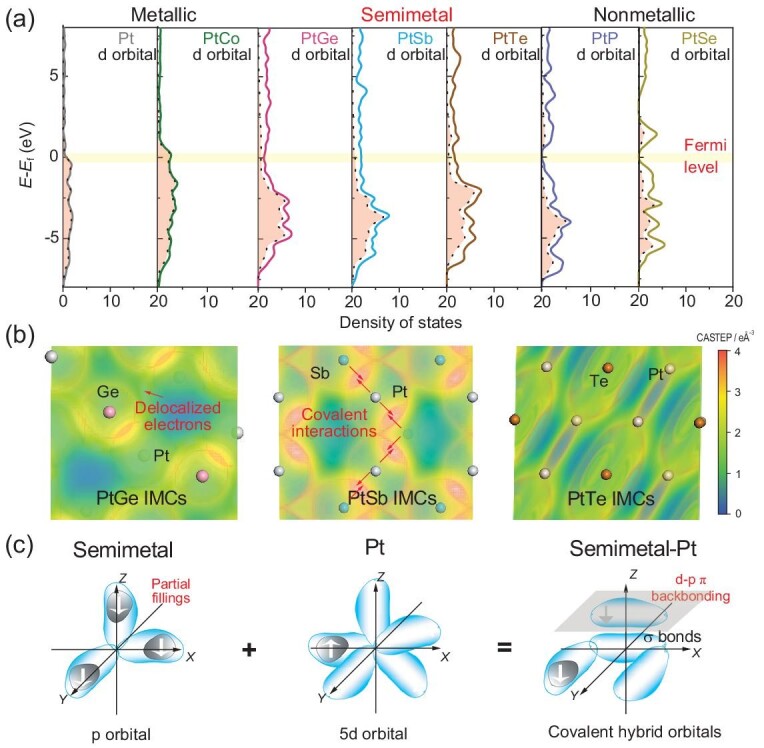
DFT predictions. (a) DFT calculations on density of states for Pt, PtCo, PtGe, PtSb, PtTe, PtP and PtSe compounds. (b) The electron distribution in semimetal-Pt IMCs with scale bar from 0 to 4 e Å^−3^. (c) The formation of d-p π backbonding as covalent interaction in semimetal-Pt IMCs.

The unique physical properties triggered by semimetal elements in Pt-based intermetallic compounds were first inspired by density functional theory (DFT) analysis. Comparisons for density of state (DOS) results among metal-, semimetal- and non-metal-Pt compounds (including Pt, PtCo, PtGe, PtSb, PtTe, PtP and PtSe) are shown in Fig. 2a. It can be noted that the semimetal-Pt compounds (PtGe, PtSb and PtTe) possess a continuous electron distribution near the Fermi level, which is similar to that of metal-Pt compounds (pure Pt and PtCo). This result indicates that semimetal-Pt compounds possess delocalized electrons, presenting as the metallic states of IMCs. For contrast, obvious vacancies could be found near the Fermi level for non-metal-Pt compounds (PtP and PtSe), which indicates that electron-localization effects lead to semiconductors for PtP and PtSe. Unconventionally, for the DOS value for surface Pt d orbitals, the Pt d orbitals in semimetal-Pt IMCs (PtGe, PtSb and PtTe) possess amounts of electrons distributed below the Fermi level from 0 to −10 eV. This property is similar to that of non-metal-Pt compounds (PtP and PtSe), but distinct from that of metal-Pt compounds (Pt and PtCo). It indicates that covalent interactions, such as electron transfer or electron share, could exist between semimetal and Pt atoms for d-orbital fillings [34]. For more direct observation of covalent interaction and metallic bonds triggered by semimetals, the electron distributions are exhibited in Fig. 2b with the charge details for specific bonds in semimetal-Pt IMCs. The scale bar from blue to red represents charge density from 0, 2 to 4 e Å^−3^. It can be observed that there is a continuous green region distributed along Pt and semimetal atoms, indicating the delocalized electron cloud. Moreover, a denser electron cloud in the red region can be clearly observed in Pt-Sb samples, which is located between Sb and Pt atoms. This increased electron distribution can be ascribed to covalent interactions from multiple bonds, such as π bonds, coordinate bonds and so on. It should also be noted that Sb atoms match well to the Pt atoms with an optimal atomic radius and energy level, so that the covalent interaction can be clearly seen between 5p orbitals in Sb, and 5d orbitals in Pt. For contrast, the atomic radius of Ge is small, while the electronegativity of Te is too high to distort the interactions. This strong covalent interaction was finally determined as d-p π backbonding based on molecular orbital theory in Fig. 2c. The semimetal atoms offer one of p orbitals to generate σ hybrid orbitals with one of d orbitals from Pt. Then electron couples of Pt perpendicular to σ bonds could transfer into empty p_z_ hybrid orbitals in semimetals, leading to d-p π backbonding as covalent interactions. Therefore, it is concluded that the strong covalent interactions, d-p π backbonding, exist in the combination of semimetal and Pt atoms with metallic states, which is expected to drive the combination of ordered Pt-M IMC bonds in synthesis.

### Synthesis of semimetal-Pt IMCs

**Figure 3.**
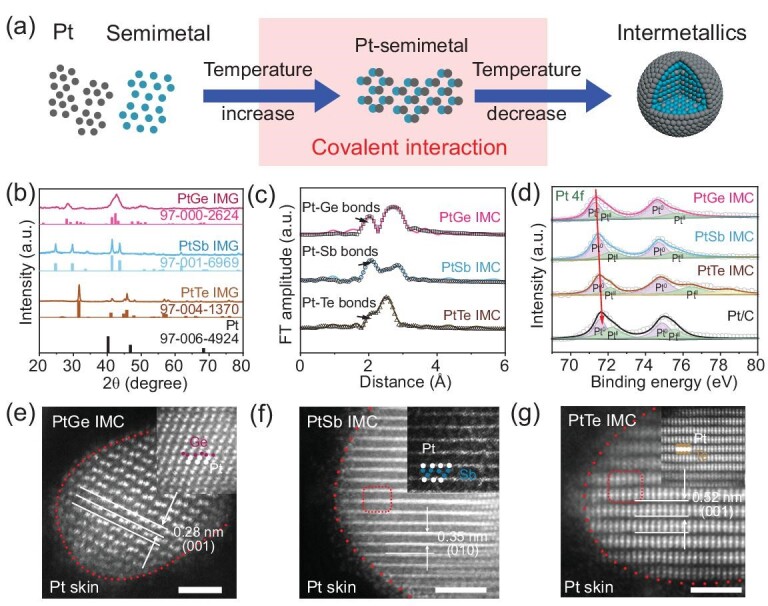
Structural analysis of as-synthesized semimetal-Pt IMCs. (a) Illustration of the synthesis process of Pt-based intermetallic structures. (b) XRD patterns for the as-synthesized PtGe, PtSb and PtTe IMC nanoparticles. (c) The r-space XAFS results and FEFFTT fitting line for the as-synthesized PtGe, PtSb and PtTe IMC nanoparticles. (d) XPS results for the as-synthesized PtGe, PtSb and PtTe IMC nanoparticles. (e) HAADF images and magnified atomic arrangements of PtGe IMC nanoparticles. (f) HAADF images and magnified atomic arrangements of PtSb IMC nanoparticles. (g) HAADF images and magnified atomic arrangements of PtTe IMC nanoparticles. The scale bar is 1 nm.

Based on the prediction of strong covalent interaction triggered by semimetal atoms, a series of semimetal-Pt intermetallic compounds such as PtGe IMC, PtSb IMC and PtTe IMC were easily synthesized with a temperature of 573 K. This temperature is 300 K lower than the existing synthetic limitation (873 K) for metal-Pt-based intermetallic compounds (as shown in Table S1), only half of the temperature limitation in Celsius (300°C for semimetal-Pt IMC and 600°C for other reported metal-Pt IMC synthesis temperatures). The mechanisms of the synthetic process triggered by covalent interaction were first proposed in Fig. 3a. As the temperature increases, the metallic bonds in Pt metals and semimetals break. Under the driving force from the strong covalent interaction of d-p π backbonding, the semimetal atoms preferentially combine into Pt atoms. Then the ordered structure of Pt intermetallic compounds reforms as the temperature decreases. X-ray diffraction (XRD) was used to verify the phase of as-prepared samples. As shown in Fig. 3b, XRD patterns of the PtGe IMC, PtSb IMC and PtTe IMC match well with the characteristic peaks in the PDF cards, which indicates the successful synthesis of semimetal-Pt IMC samples. X-ray absorption fine structure spectroscopy (XAFS) was also performed to investigate the structural information of the synthesized samples. In Fig. 3c, Pt-Ge, Pt-Sb and Pt-Te bonds can be clearly observed in each sample, with the FEFFTT fitting line corresponding to the standard structure of semimetal-Pt IMCs (see the fitting parameters and scattering paths in Fig. S2). To determine the electronic state of Pt sites in semimetal-Pt IMCs, X-ray photoelectron spectroscopy (XPS) was employed to measure the valence of Pt in Fig. 3d. It can be observed that the binding energy of Pt (0) in the Pt/C is the highest among all the samples, and that of the PtGe IMC is the lowest, corresponding to the electronegativity sequence of Ge < Sb < Te ∼ Pt. It is noted that a Pt (IV) peak can be observed in the PtTe IMC, which is ascribed to the highest electronegativity and oxytropism of Te elements. The Pt content of as-synthesized semimetal-Pt IMCs is also determined by inductively coupled plasma optical emission spectrometry (ICP-OES) (Table S2); the mass ratio of Pt is 0.23 (PtGe IMC), 0.19 (PtSb IMC) and 0.22 (PtTe IMC), respectively. Structural morphology of prepared semimetal-Pt IMCs with Pt skin was characterized by transmission electron microscopy (TEM) and high-angle annular dark-field scanning transmission electron microscopy (HAADF-STEM) images. In Fig. S3 (TEM images of semimetal-Pt IMCs), the PtGe IMC, PtSb IMC and PtTe IMC samples present as nanoparticles with a size of ∼5 nm (Fig. S4, the size distribution of IMCs). For atomic arrangement of the PtGe IMC in Fig. 3e, regular light or dark alignment can be clearly observed in the particles. The light dots represent Pt atoms and relatively darker dots represent semimetal atoms. Clear and uniform atomic arrangement can be observed, which indicates the highly ordered crystalline degree of semimetal-Pt IMC samples triggered by covalent interactions. For the PtSb IMC in Fig. 3f and PtTe IMC in Fig. 3g, ordered arrangement between semimetal atoms and Pt atoms can also be directly observed. All the above results demonstrate that semimetal-Pt intermetallic particles (such as PtGe IMC, PtSb IMC and PtTe IMC) were easily synthesized based on covalent interactions.

### Electrocatalytic activity and CO tolerance

**Figure 4.**
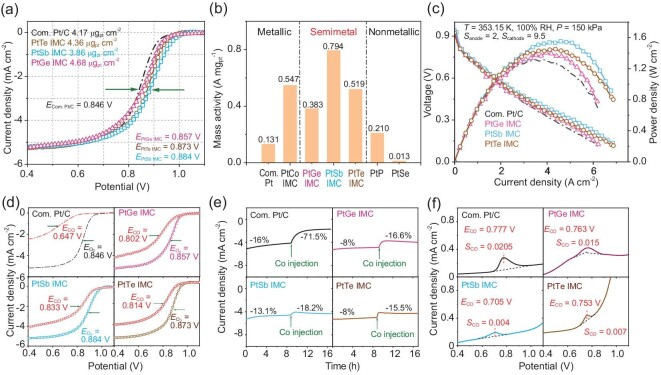
(a) ORR polarization curves for the as-synthesized semimetal-Pt IMCs and commercial Pt/C. (b) The mass activity of as-prepared metal-, semimetal- and non-metal-Pt compounds. (c) H_2_-O_2_ fuel-cell tests for as-synthesized semimetal-Pt IMCs and commercial Pt/C. (d) ORR polarization curves for as-synthesized semimetal-Pt IMCs and commercial Pt/C in 100 ppm CO poison. (e) The *i*-*t* curves for as-synthesized semimetal-Pt IMCs in 100 ppm CO poison. (f) The CO stripping tests for as-synthesized semimetal-Pt IMCs.

To verify the potential advantages of semimetal-Pt IMCs (metallic states and covalent interactions), as-prepared semimetal-Pt IMCs with Pt skin serve as electrocatalysts in half-cell and fuel-cell measurements. The oxygen reduction reaction (ORR) polarization curves for as-synthesized semimetal-Pt IMCs and commercial Pt/C are recorded in Fig. 4a. The loading of electrocatalysts is ∼0.02 mg cm^−2^, and the Pt loading is further calculated to be ∼4 μg cm^−2^ for each electrode. The half-wave potential for PtSb, PtTe and PtGe IMCs is 0.884 V, 0.873 V and 0.857 V, respectively. The PtSb IMC possesses the lowest overpotential, which is 38 mV lower than that of commercial Pt/C. The mass activities of the as-prepared metal-, semimetal- and non-metal-Pt compounds are also compared in Fig. 4b, and Figs S10 and S13 (ORR results for Pt/C, PtCo IMC, PtGe IMC, PtSb IMC, PtTe IMC, PtP and PtSe compounds). The semimetal-Pt IMCs reach a high mass activity of 0.794 A mg^−1^ for PtSb, 0.519 A mg^−1^ for PtTe and 0.383 A mg^−1^ for PtGe. The mass activity of PtSb is more than six times that of commercial Pt/C, reaching the top value of reported electrocatalysts. The cyclic stability tests were also performed to verify the durability of semimetal-Pt IMCs. As shown in Fig. S9, ∼48 mV decay of half-wave potential can be observed for commercial Pt/C after 20 000 cycles. As for the semimetal-Pt IMCs, the potential decay is 31 mV for PtGe IMC, 37 mV for PtSb IMC and 25 mV for PtTe IMC. This indicated that a considerable durability can be observed in semimetal-Pt IMCs, which can be contributed by strong covalent interaction with stable chemical bonds and intermetallic ordered structures. For more practical applications, semimetal-Pt IMCs were employed to serve as cathodic electrocatalysts in H_2_-O_2_ fuel cells. As shown in Fig. 4c, the PtSb IMC also exhibits the highest activity; this trend is similar to that of the ORR tests. The peak power density for a PtSb-IMC-based fuel cell can reach a value of 1.57 W cm^−2^, which is almost 370 mW cm^−2^ higher than that of commercial Pt/C. The above results clearly show that semimetal-Pt IMCs possess high electrocatalytic activity for ORR and fuel cells.

The CO tolerance for as-prepared electrocatalysts was also estimated. The 100 ppm CO/O_2_ mixture was used as fuel to show the anti-poison effect in the ORR. It can be observed that semimetal-Pt IMCs exhibit outstanding CO tolerance in Fig. 4d. The decay of half-wave potential is only 51 mV for the PtSb IMC (0.833 V in 100 ppm CO poison and 0.884 V in pure O_2_), 59 mV decay for PtTe IMC, 55 mV for PtGe IMC, but 199 mV for commercial Pt/C. The constant-V CO tolerance for each sample was also investigated, as shown in Fig. 4e. All semimetal-Pt IMCs drop less than 10% after 100 ppm CO injection, while commercial Pt/C drops 55.5% (71.5% of total decay and 16% without CO injection). The CO tolerance for PtSb (5.1% decay) is more than 11 times better than that of commercial Pt/C, which is the highest CO tolerance ability among Pt-based electrocatalysts (Table S3). It should be noted that slight improvements in stability can be observed (less current decay without CO injection), which can be ascribed to the stable chemical bonds from strong covalent interaction in semimetal-Pt IMCs. To seek direct evidence for CO tolerance for semimetal-Pt IMCs, CO stripping tests were also performed. As shown in Fig. 4f, the CO adsorption peak of commercial Pt/C is located at 0.777 V with an area of 0.0205 mW cm^−2^ (with the same scan rate and Pt loading for each electrode, the area of CO adsorption peak can represent the CO-poison electrochemical surface area). For the semimetal-Pt IMCs, the peaks shifted more negatively (0.705 V for PtSb, 0.753 V for PtTe and 0.763 V for PtGe) and the area also decreased to 0.004 mW cm^−2^ for the PtSb IMCs. These results indicate the easier desorption and oxidation of CO molecules on catalysts’ surface, indicating the high CO tolerance of semimetal-Pt IMCs. Therefore, all of the above results demonstrate that the covalent interaction in metallic semimetal-Pt IMCs not only benefits synthesis, but could also contribute to high mass activity and CO tolerance in fuel-cell electrocatalysis.

### Mechanism understanding

**Figure 5.**
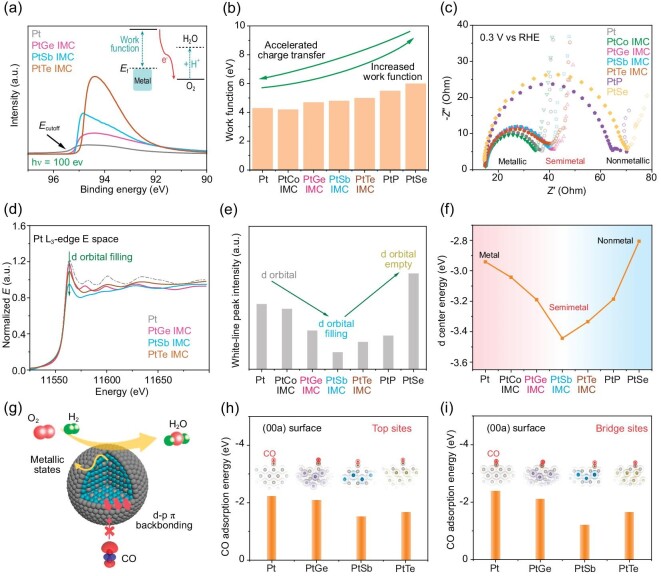
(a) UPS spectra of semimetal-Pt IMCs and commercial Pt/C. (b) The calculated work function of as-prepared metal-, semimetal- and non-metal-Pt compounds. (c) EIS analysis of all the as-prepared samples. (d) The near-edge XAFS results for as-synthesized semimetal-Pt IMCs. (e) The intensity of the white-line peak for all prepared samples. (f) The calculated d-center energy of the as-prepared metal-, semimetal- and non-metal-Pt compounds. (g) Scheme illustration for enhanced ORR activity and CO tolerance in semimetal-Pt samples. (h) CO adsorption energy of the top sites on Pt atoms for pure Pt and semimetal-Pt IMCs with Pt skin. (i) CO adsorption energy of the bridge sites.

To further verify the function of covalent interactions and metallic states in semimetal-Pt IMCs, more in-depth characterizations were performed. Ultraviolet photoelectron spectroscopy (UPS) in Fig. 5a and Fig. S17 (UPS for all Pt-based compounds) was employed to investigate metallic properties of semimetal-Pt bonds. Based on the UPS results, the work functions of metal-, semimetal- and non-metal-Pt compounds are also compared in Fig. 5b. The work function value of semimetal-Pt IMCs is similar to that of metal-Pt and smaller than that of non-metal-Pt. This indicates that the electrons can easily transfer within electrocatalysts and couple to surface sites in semimetal-Pt IMCs (Fig. 5a inset). The electrochemical impedance spectroscopy (EIS) analysis at non-catalytic regions of all-prepared compounds also confirms the accelerated charge transport in Fig. 5c [35]. The semicircles of PtGe, PtSb and PtTe are near those of the Pt and PtCo samples, which are much smaller than those of PtP and PtSe. This result corresponds to the UPS analysis, in which the semimetal-Pt IMCs promote fast charge transport as metallic states. It could be the main reason for enhanced electrocatalytic mass activity of semimetal-Pt IMCs compared to non-metal-Pt compounds (Fig. 5g). On the other hand, the covalent interactions of semimetal-Pt bonds were also demonstrated. As shown in Fig. 5d and Fig. S18 (XAFS for Pt-based compounds), the density of white-line peak decreased from pure Pt to the PtSb IMC in Pt L_3_-edge (a lower white-line peak density represents more d-orbital fillings). The white-line peak density of semimetal-Pt IMCs is relatively lower than that of metal-based Pt (Fig. 5e), which indicates that more electrons are filling in d orbitals for semimetal-Pt samples (the sharp increase in PtSe can be attributed to the high electronegativity of Se). Moreover, the d-center energy was also calculated in Fig. 5f based on DOS results to verify the d-orbital fillings (as shown in the Methods section of the Supplementary data). It is well known that more d-orbital fillings can directly lead to the downshift of the d-band center. The semimetal-Pt IMCs and PtP possess a relatively lower d-band center than pure Pt. The PtSb IMC has the lowest d-center energy of −3.46 eV, corresponding to the XAFS value of d-orbital fillings. All the results of d-orbital fillings match with the results of DOS and electron distribution in Fig. 2, indicating the increased localized electrons of Pt atoms from strong covalent interactions. Therefore, the CO tolerance in semimetal-Pt IMCs can be further explained based on molecular orbital theory (Fig. S1, DOS results for CO adsorption). As semimetal-Pt bonds possess strong covalent interaction for more d-orbital fillings, the d-band center of Pt will downshift and decrease the overlap from CO 2π* orbitals, resulting in weaker CO adsorption (Fig. 5g). To verify this speculation, the CO adsorption energy on the Pt surface of semimetal-Pt IMCs was also calculated. As shown in Fig. 5h and i, it is obvious that the CO adsorption energy on the Pt skin of semimetal-Pt IMCs is lower than that of pure Pt, both for CO adsorbed top sites and bridge sites. The semimetal-Pt IMCs have relatively weaker CO adsorption than metal-Pt compounds, leading to considerable CO tolerance ability. Therefore, all of the above results clearly predict that covalent interaction and metallic states can be combined in semimetal-Pt IMCs, which benefits CO-tolerant fuel cells.

## CONCLUSION

In this work, we report that the covalent interactions in semimetal-Pt intermetallic compounds can promote both low-temperature synthesis and fuel-cell electrocatalysis. The DFT and synchrotron results demonstrated that the covalent interactions in semimetal-Pt IMCs are ascribed to d-p π backbonding, contributing to recombination of ordered Pt-M metallic bonds and orbital fillings of active sites. Our as-prepared semimetal-Pt IMCs were obtained with a temperature 300 K lower than the synthesis temperature for other metal-Pt IMCs, and the CO-tolerant activity is 11 times better than commercial Pt/C, reaching the highest value among reported electrocatalysts. We anticipate that the covalent interaction in semimetal-Pt IMCs can offer new insights into the rational synthesis of intermetallic structures and produce advanced electrocatalysts for practical fuel cells.

## MATERIALS AND METHODS

All the chemical reagents were purchased from Aladdin Reagent Corporation Ltd. Synthesis procedures, materials characterization, electrochemical tests, fuel-cell measurements and computational methods can be found in the Methods section of the Supplementary data.

## Supplementary Material

nwae233_Supplemental_File
