# Supplementary material for: Semimetal-triggered covalent interaction in Pt-based intermetallics for fuel-cell electrocatalysis
Source: Natl Sci Rev. 2024 Jul 8;11(8):nwae233. doi: 10.1093/nsr/nwae233 (PMC11308177; doi:10.1093/nsr/nwae233)
Supplement: nwae233_Supplemental_File [file nwae233_supplemental_file.docx]

Supplementary Information for

Semimetal-triggered Covalent Interaction in Pt-based Intermetallics for Fuel-cell Electrolysis

Han Cheng,^1^† Renjie Gui,^1^† Chen Chen,^2^† Si Liu,^3^ Xuemin Cao,^1^ Yifan Yin,^1^ Ruize Ma,^1^ Wenjie Wang,^2^ Tianpei Zhou,^1^ Xusheng Zheng,^2^ Wangsheng Chu,^2^ Yi Xie,^14^ and Changzheng Wu^14^*

Changzheng Wu

Email: [czwu@ustc.edu.cn](mailto:czwu@ustc.edu.cn)

**This file includes:**

Methods

Figs. S1 to S19

Table S1 to S3

**Methods：**

***Synthesis procedures***

**Synthesis of PtGe/PtSb/PtTe IMCs.** 15 mg H_2_PtCl_6_ 6H_2_O were dissolved in 4 mL of ethanol. Then the 10 mg of tetrahexylammonium bromide was added as ligands to cross-link metal ions and carbon support. The solution was stirring for 10 min and following added 30 mg of Ketjenblack-600 carbon. The mixture was under ultrasonication for 30 min and further lyophilization. After that, the dried solid was annealed at Ar atmosphere at 573 K for 2 h to obtain Pt nanoparticles-KJ. Then excess GeCl_4_/SbCl_3_/Te were put in another porcelain boat at the upstream Ar. The porcelain boat was annealing at furnace at 573 K for 2 h in Ar flow.

**Synthesis of PtP / PtSe compounds.** 15 mg H_2_PtCl_6_ • 6H_2_O were dissolved in 4 mL of ethanol. Then the 10 mg of tetrahexylammonium bromide was added as ligands to cross-link metal ions and carbon support. The solution was stirring for 10 min and following added 30 mg of Ketjenblack-600 carbon. The mixture was under ultrasonication for 30 min and further lyophilization. After that, the dried solid was annealed at Ar atmosphere at 573 K for 2 h to obtain Pt nanoparticles-KJ. Then excess NaH_2_PO_2_ and Se Powder were put in another porcelain boat at the upstream Ar. The porcelain boat was annealing at furnace at 573 K for 2 h in Ar flow.

**Synthesis of PtCo IMC (intermetallic compounds).** H_2_PtCl_6_ • 6H_2_O was selected as Pt source and CoCl_2_ • 6H_2_O was selected as Co source respectively. 15 mg H_2_PtCl_6_ • 6H_2_O and 13.68 mg CoCl_2_ • 6H_2_O were dissolved in 4 ml of ethanol. Then the 10 mg of tetrahexylammonium bromide was added as ligand to cross-link metal ions and carbon support. The solution was stirring for 10 min and following added 30 mg of Ketjenblack-600 carbon. The mixture was under ultrasonication for 30 min and further lyophilization. After that, the dried solid was grind and finally annealed at Ar atmosphere at 873 K for 2 h.

**Synthesis of Pt skin on Pt based IMC.** All the obtained samples above was taken for 100 mg and dispersed into 10% HNO_3_ solution. The mixture was keeping at 333 K for 30 min. The the solid was washed by H_2_O for three times and centrifuged collection. The dried solid was finally annealed at Ar atmosphere at 573 K for 2 h to fabricate Pt skin.

***Materials characterization***

The obtained samples were first analyzed by X-ray powder diffraction instrument (Philips X’ Pert Pro Super diffractometer), in which the radiation source is Cu Ka with 1.54178 Å of λ value. The X-ray photoelectron spectra (XPS) experiments were performed in ESCALAB MK II X-ray photoelectron spectrometer with Ar ion gun equipped. The morphology of sample was examined by transmission electron microscope JEM-2100F field-emission electron (TEM) and JEOL JEM-ARF200F atomic resolution analytical microscope for high-angle annular dark-field scanning transmission electron microscopy image. The X-ray absorption fine structure (XAFS) were performed at the BL14W1 beamline in Shanghai Synchrotron Radiation Facility (SSRF), China. The operation protocol for HAADF-STEM (high-angle annular dark field scanning transmission electron microscope) is similar with the transmission electron microscope.

***Electrochemical tests***

The samples were mixed with 1 ml of water and 1ml of ethanol solution with a serum bottle. 5% Nafion solution was adapted as proton conductor and cross-linking agent and the mixed ink was further prepared through ultrasonic dispersion for 60 min. Combined with ICP results, about 8 μl ink was used for spray coated on glassy carbon and the loading of each electrocatalyst was determined as 0.02 mg/cm^2^. The Pine ASR instrument and CHI760D electrochemical station were used for half-cell tests. Luggin capillary-based electrochemical cells were used. 0.1 M HClO_4_ was selected as the electrolyte and oxygen was blew to saturated solution for oxygen reduction reaction. Ag/AgCl electrode and graphite rod were used as reference and counter electrodes, respectively. The chronoamperometric measurements with constant electrochemical potential V were performed. The potential is selected at 0.5 V vs RHE, which is located at limiting diffusion current region. The electrolyte was selected as 0.1 M HClO_4_ with continuous O_2_ flow bubbling. The counter electrode is graphite rod and Ag/AgCl was employed as reference electrodes with rotating speed is 1600 rpm. After i-t curve recording for 9h, the O_2_ flow was changed to the mixture gas (100 ppm CO in O_2_). All the other parameter keep unchanged such as potential and rotating speed. The CO poison tests were performed under the atmosphere of O_2_ with 100 ppm CO mixture gas. The CO adsorption procedure in CO-stripping voltammogram measurements was accomplished by polarizing the electrode at 0.2 V with CO bubbling in electrolyte solution for 10 min to adsorb monolayer CO molecules. Then, the electrode was transferred to another cell filled with Ar-saturated 0.1 M HClO_4_ solution. Then, cyclic voltammograms (CVs) were conducted from 0.05 to 1.2 V with a scan rate of 50 mV/s. The theoretical charge per unit area was used as Q_theo, Pt_ = 420 mC/cm^2^ and corrected for capacitive contributions (the 2nd cycle).

***Fuel-cell measurements***

The platinum loading of all catalysts for the cathode was about 0.2 mg/cm^2^. The anode catalyst was 60 wt%Pt/C, and the loading was also 0.2 mg/cm^2^. Catalyst ink was prepared by ultrasonic dispersion of catalyst (1.4 to 1.6 mg) and 5 weight (wt)% Nafion solution in 1.0 mL deionized water for 1 h. Then ink was sprayed on Ballard avcarb GDS3250 carbon paper (1.2*1.2 cm^2^) pretreated with PTFE and dry with an infrared lamp. Then the membrane electrode assembly (MEA) was prepared by hot pressing method with Gore proton exchange membrane M82015. Relative pressure is about 3 MPa for 180 s. Then the MEA was package in single cell and tested in 850e fuel cell test system (Scribner associates Inc.). The semimetal-Pt IMCs and commercial Pt/C based MEA was tested at 353.15 K (80 °C) with 100% RH using the 850e fuel-cell test system (Scribner Associates Inc.). The total outlet pressure was adjusted to 150 kPa with the stoichiometric flow rates of anode (*s* = 2) and cathode (*s* = 9.5 for O_2_). Before the polarization curves were recorded, the MEA was fully activated by holding at 0.9 V for 20 min and 0.7 V for 20 min to stabilize potential and current density.

***Computational methods***

All the calculations are performed in the framework of the density functional theory with the projector augmented plane-wave method, as implemented in the Vienna ab initio simulation package. The generalzied gradient approximation proposed by Perdew, Burke, and Ernzerhof is selected for the exchange-correlation potential. The long-range van der Waals interaction is described by the DFT-D3 approach. The cut-off energy for plane wave is set to 450 eV. The energy criterion is set to 10−5 eV in iterative solution of the Kohn-Sham equation. A vacuum layer of 15 Å is added perpendicular to the sheet to avoid artificial interaction between periodic images. All the structures are relaxed until the residual forces on the atoms have declined to less than 0.03 eV/Å. The d-center energy of all the samples was performed as below. The PDOS diagrams of d orbitals were calculated based on Pt atoms. Then, the density of states is integrated by energy levels from negative infinity to the Fermi level to obtain the number of d electrons occupying the orbitals. After that, multiply the energy level and state density, the value is following integrated by energy levels to obtain the total energy of the electrons occupying d orbital. Finally, divide the total energy by the number of electrons to get the average energy of d electrons, which is the value of d-band center.


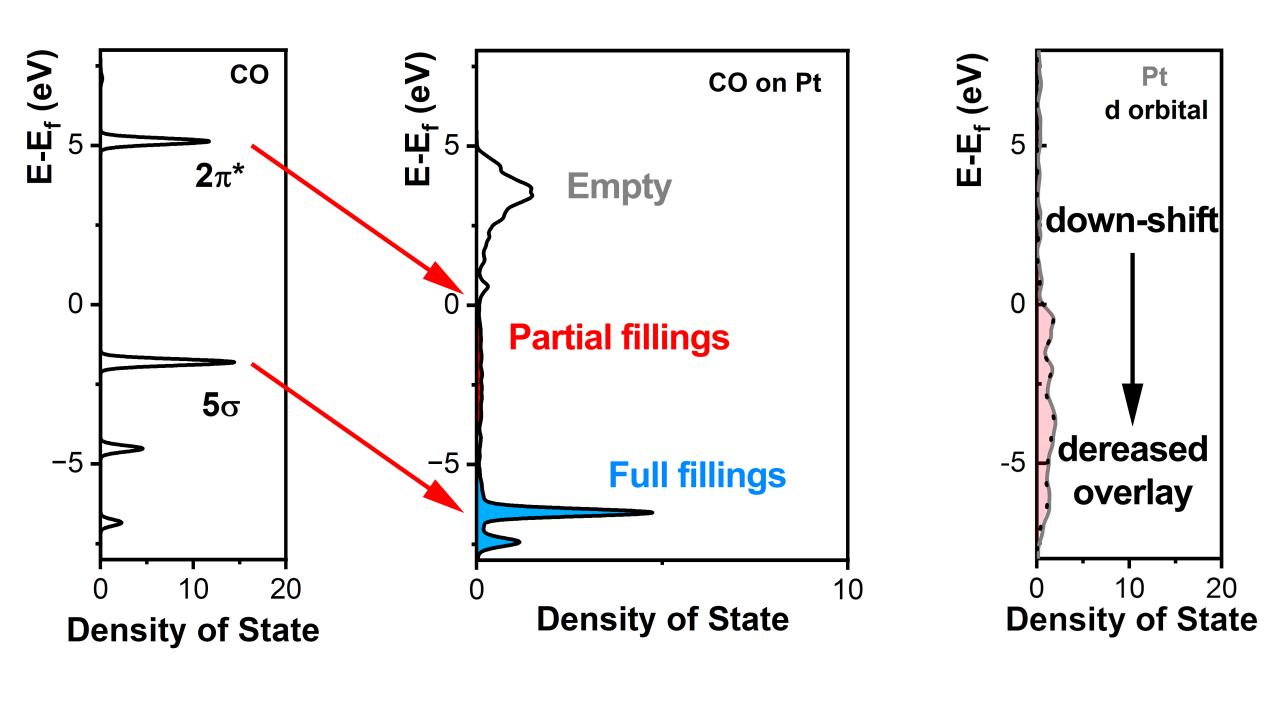
**S1. DOS results for CO molecules adsorption**

**Figure S1.** DOS results for CO molecules adsorption.

As the CO molecule adsorbed on the Pt surface, the 5σ and 2π* orbitals expanded more broadly and shifted down. Partial electrons in the Pt d band were fed back, filling in the CO 2π* orbitals (below the Fermi level part). Most of the 5σ orbitals were located below the Fermi level and occupied by electrons. The electrons fed back from Pt d band could only fill into the 2π* orbitals (below the Fermi level part). Therefore, the downshift of the Pt d band can decrease the overlay with CO orbitals.

**S2. Fitting parameters and scattering paths for XAFS**

**(a)**


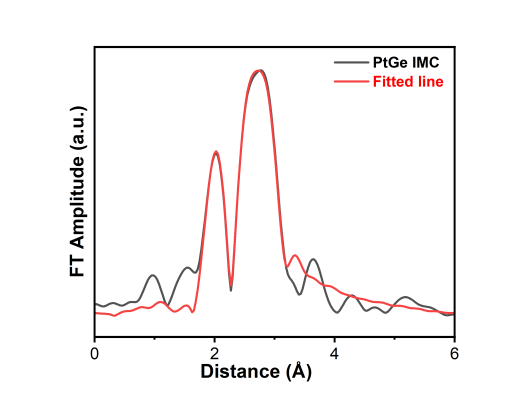

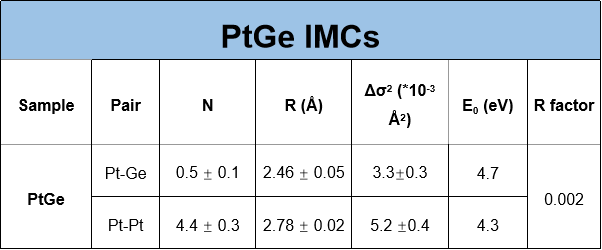


**(b)**


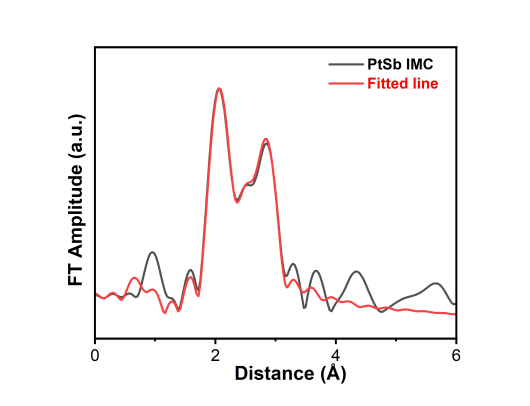

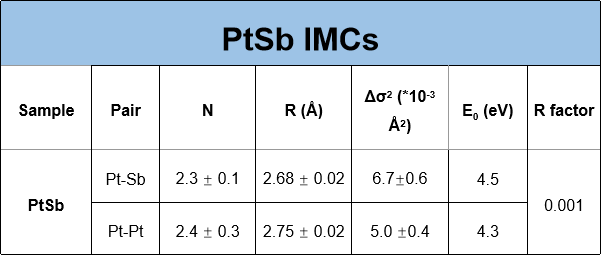


**(c)**


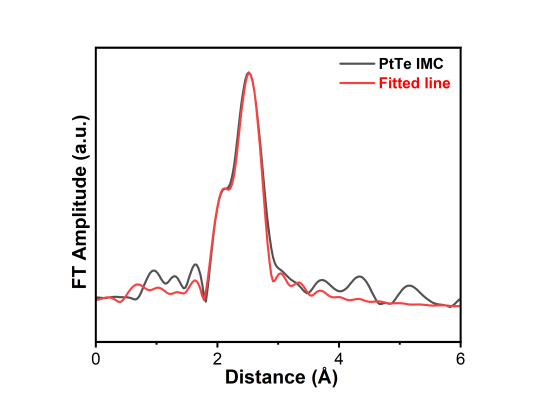

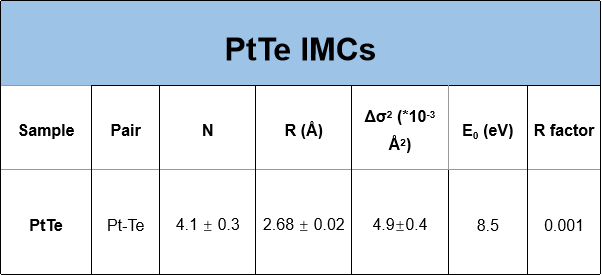


**Figure S2.** (a) The r-space of XAFS results with fitted line (left) and fitting parameters with different scattering paths (right) of PtGe IMCs. (b) The r-space of XAFS results with fitted line (left) and fitting parameters with different scattering paths (right) of PtSb IMCs. (c) The r-space of XAFS results with fitted line (left) and fitting parameters with different scattering paths (right) of PtTe IMCs.

**S3.** **TEM images of semimetal-Pt intermetallics**

**(c)**

**(b)**

**(a)**


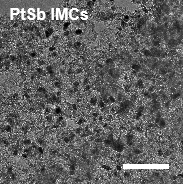

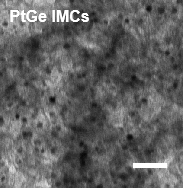

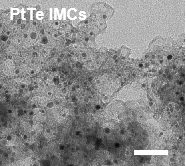


**Figure S3.** The TEM images of semimetal-Pt intermetallic (a) for PtGe IMC. (b) for PtSb IMC. (c) for PtTe IMC.The scale bar is 100 nm.

**S4. The size distribution of IMCs.**

**(c)**

**(a)**

**(b)**


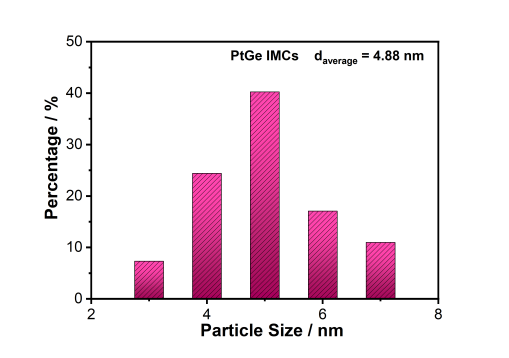

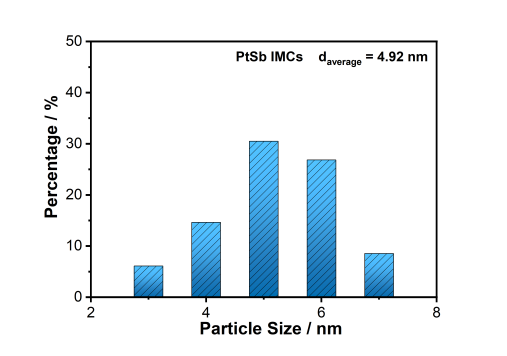

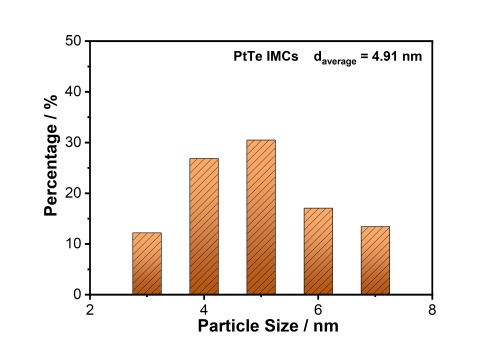


**Figure S4.** The size distribution for PtGe IMCs (a), PtSb IMCs. (b) and PtTe IMCs (c).

**S5. The lattice structure of semimetal-Pt intermetallics**


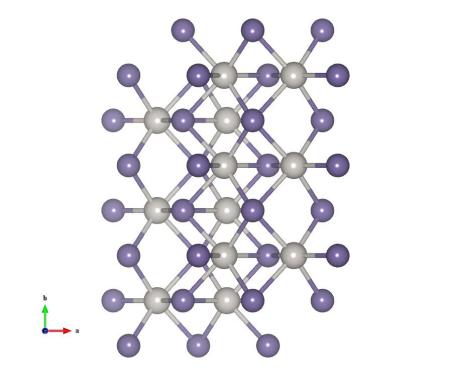


**(a)**


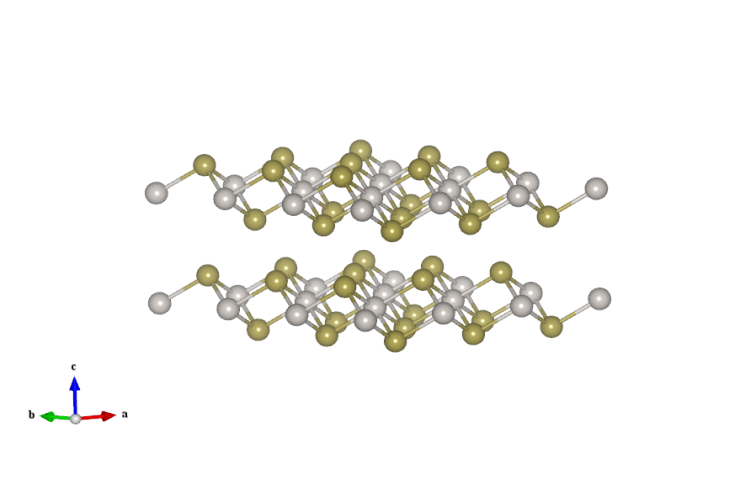

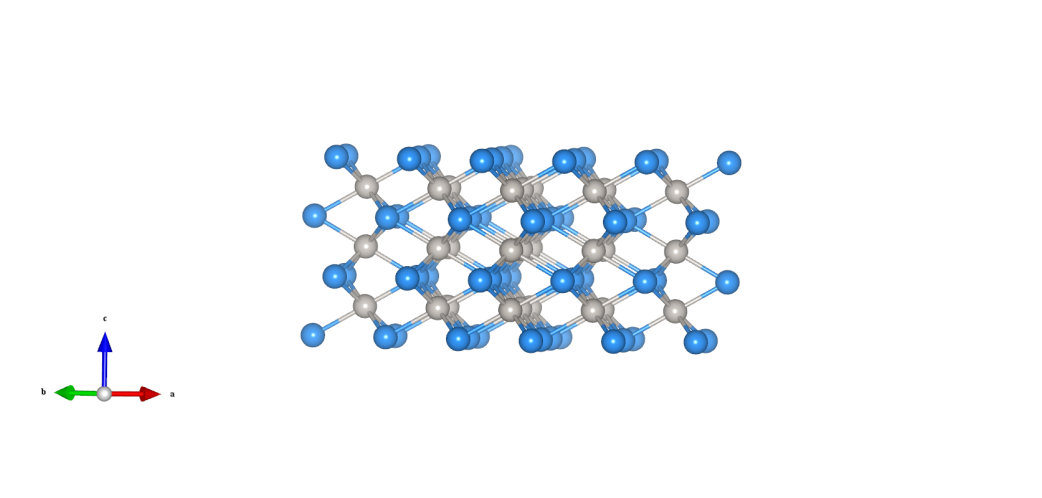


**(c)**

**(b)**

**Figure S5.** The lattice structure of semimetal-Pt intermetallics. (a) for PtGe IMC. (b) for PtSb IMC. (c) for PtTe IMC.

**S6. EDS mapping images of semimetal-Pt intermetallics**

**(a)**

**(b)**


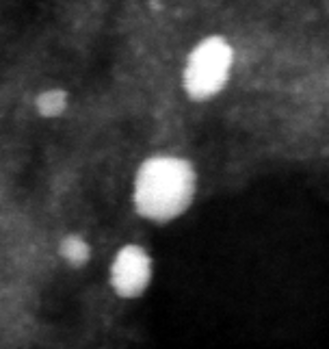

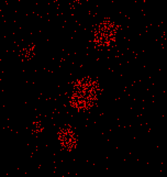

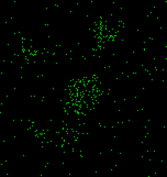


**Pt**

**Ge**


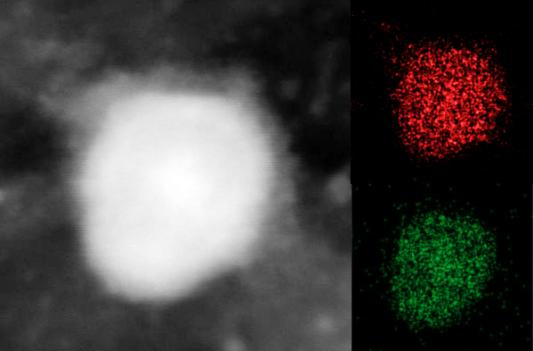


**Pt**

**Sb**

**(c)**


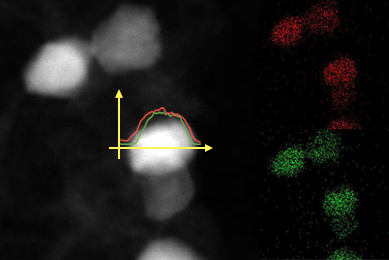


**Pt content**

**Te content**

**Pt**

**Te**

**Figure S6.** The EDS mapping images of semimetal-Pt intermetallics. (a) for PtGe IMC. (b) for PtSb IMC. (c) for PtTe IMC. The scal bar is 4 nm.

**S7. HRTEM images of semimetal-Pt intermetallics**

**(b)**

**(c)**

**(a)**


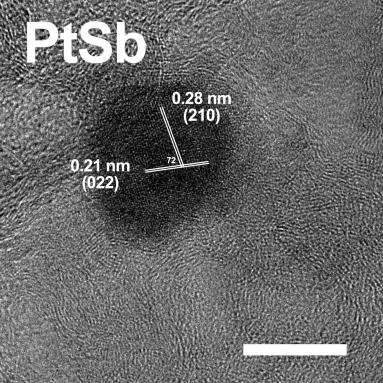

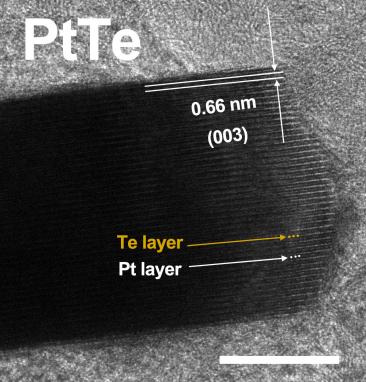

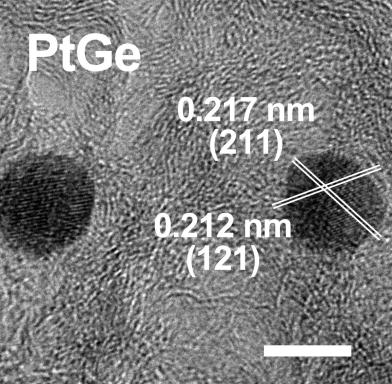


**Figure S7.** The HRTEM images of semimetal-Pt intermetallics. (a) for PtGe IMC. (b) for PtSb IMC. (c) for PtTe IMC. The scale bar is 4 nm.

**S8. ORR tests for different Pt loading of commercial Pt/C**


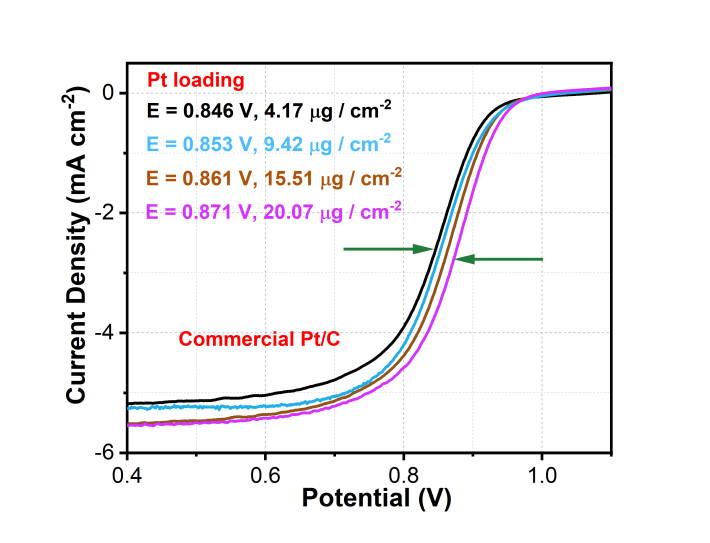


**Figure S8.** The ORR tests for different Pt loading of commercial Pt/C from 4.17 μg/cm^-2^ to 20.07 μg/cm^-2^.

**S9. Cyclic stability tests for electrocatalysts**


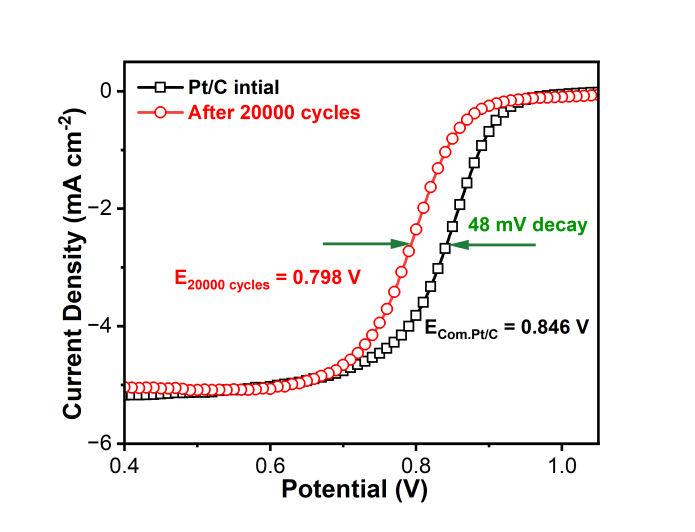

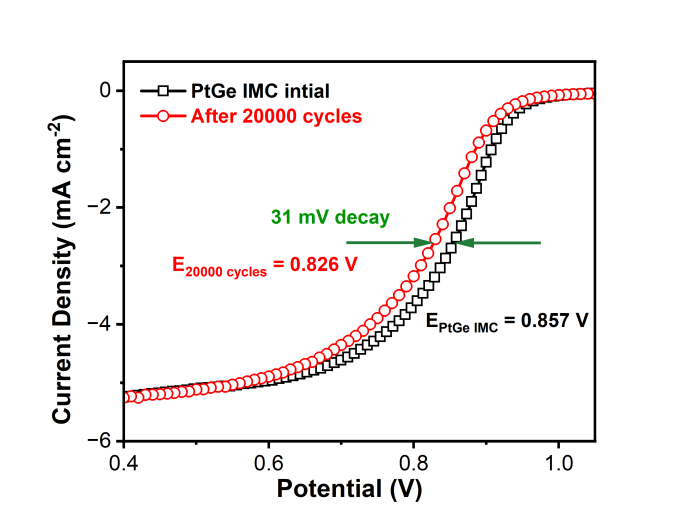


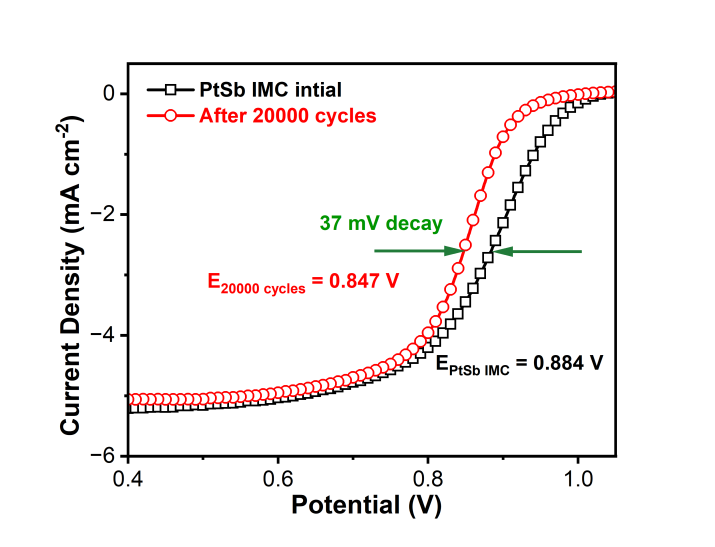

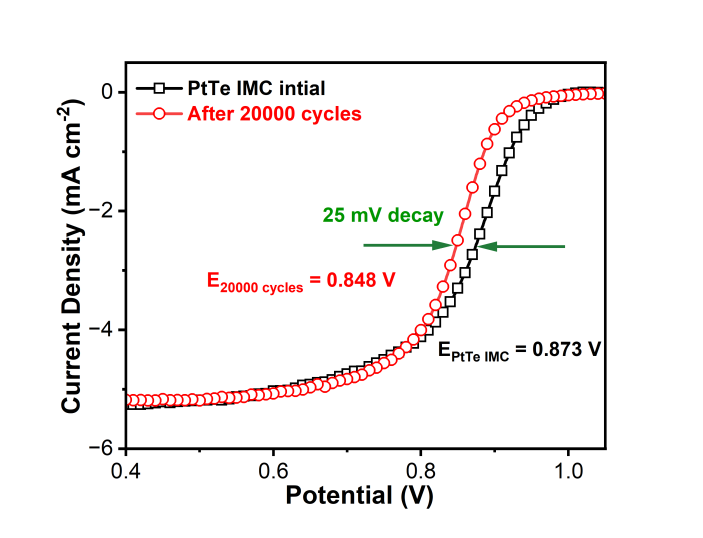


**Figure S9.** The cyclic stability tests for commercial Pt/C (a), PtGe IMC (b), PtSb IMC (c) and PtTe IMC (d).

**S10. ORR curves for PtP and PtSe compounds.**


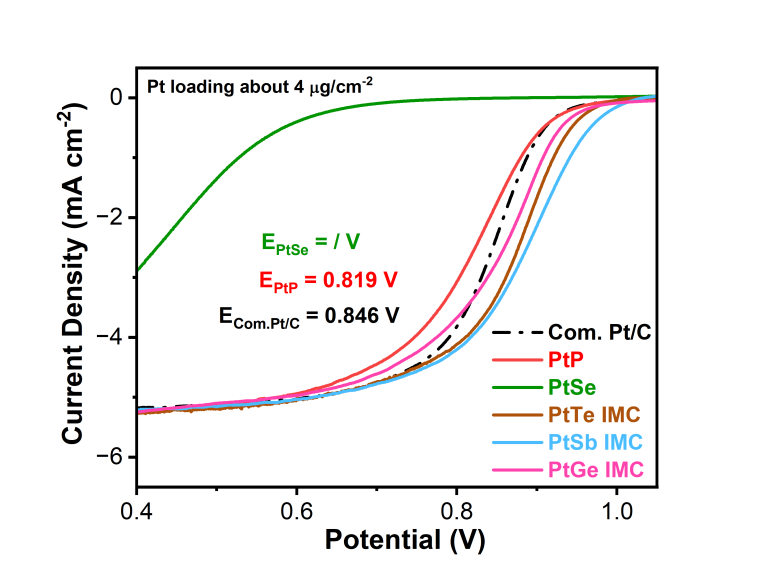


**Figure S10.** ORR curves for PtP and PtSe compounds.

**S11. Fuel-cell stability tests.**


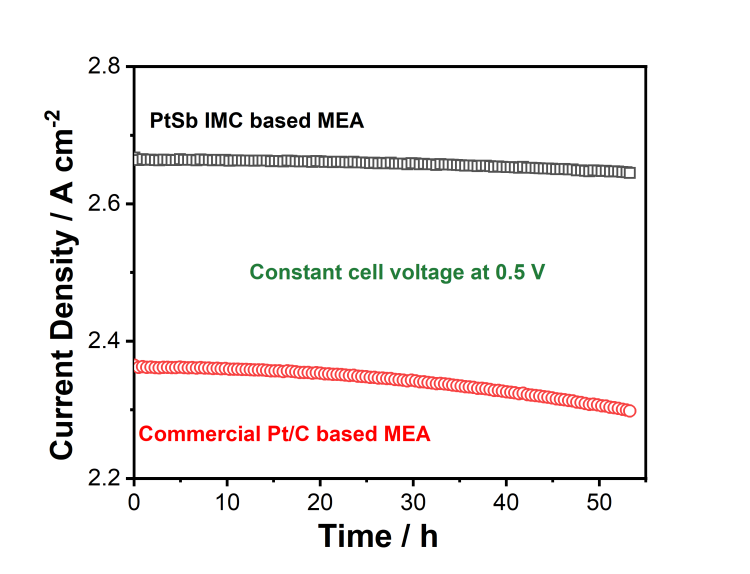


**Figure S11.** The chronoamperometric tests for commercial Pt/C and PtSb IMC in fuel cells.

To verify the stability of fuel cells, the chronoamperometric measurements were performed both for semimetal-Pt IMCs and commercial Pt/C. The constant cell voltage of 0.5 V was hold for durability measurements in H_2_/O_2_ environment with 150 kPa outlet pressure and stoichiometric flow rates (anode *s* = 2, cathode *s* = 9.5). As shown in **Figure S11**, the semimetal-Pt IMCs exhibit considerable durability compared to commercial Pt/C. Similarly, only slight improvement of stability can be observed for PtSb IMCs, which can be ascribed to the stable chemical bonds from strong covalent interaction in semimetal-Pt IMCs.

**S12. Characterization for PtCo IMC samples**

**(b)**

**(c)**

**(a)**


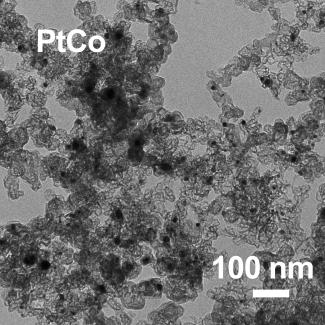

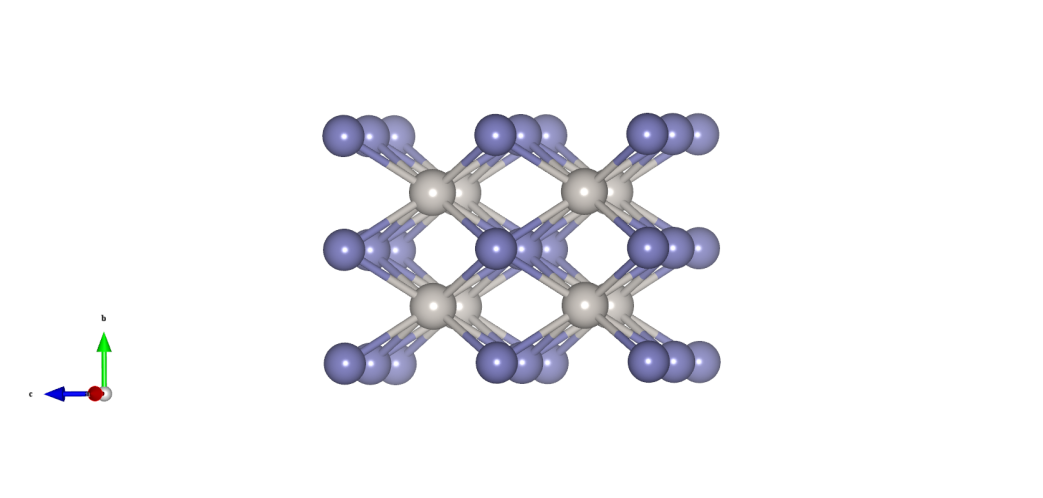

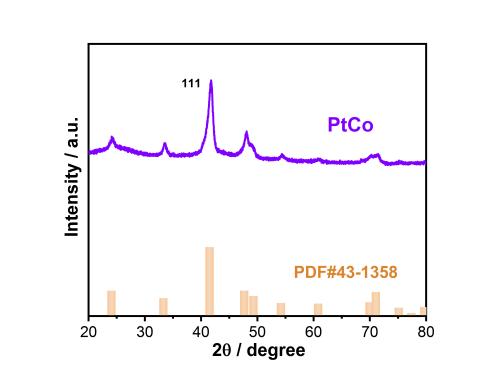


**(d)**

**(e)**

**(f)**


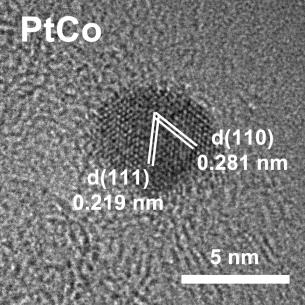

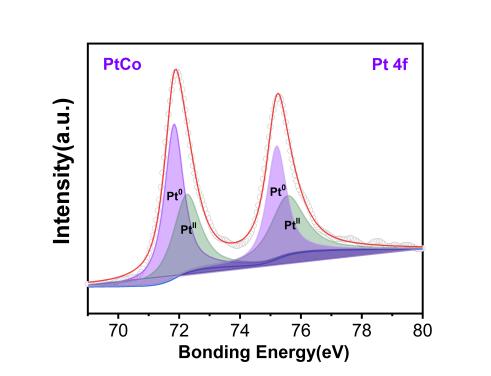

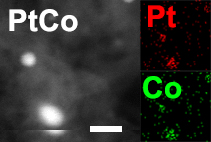


**Figure S12.** (a) The structure of PtCo IMCs. (b) The XRD patterns of PtCo IMCs. (c) TEM images of PtCo IMCs. (d) HRTEM images of PtCo IMCs. (e) EDS mapping of PtCo IMCs. (f) XPS of PtCo IMCs.

**S13. ORR and fuel cell activity for PtCo IMCs.**

**(b)**

**(a)**


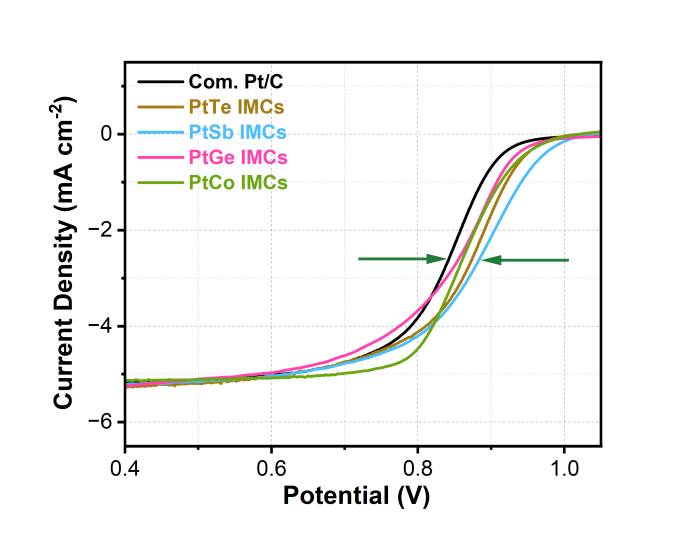

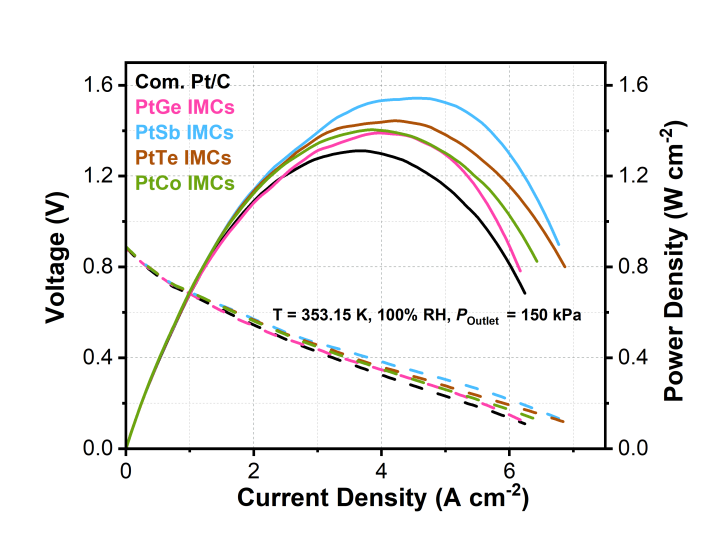


**Figure S13.** (a) ORR polarization curves for the as-synthesized semimetal-Pt IMCs and PtCo IMC. (b) H_2_-O_2_ fuel-cell tests for as-synthesized semimetal-Pt IMCs and PtCo IMC.

**S14. CO tolerance for PtCo IMCs.**


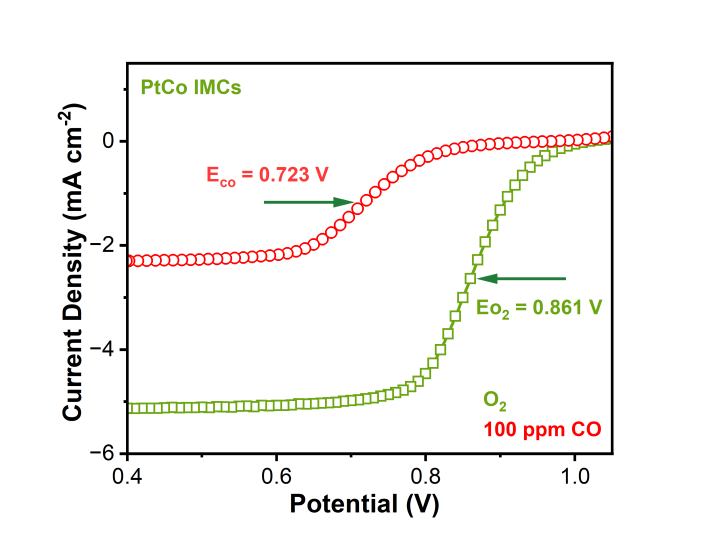


**Figure S14.** CO tolerance tests for PtCo IMC.

**S15. Characterization for PtP samples**

**(c)**

**(b)**

**(a)**


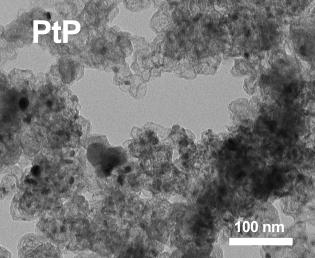

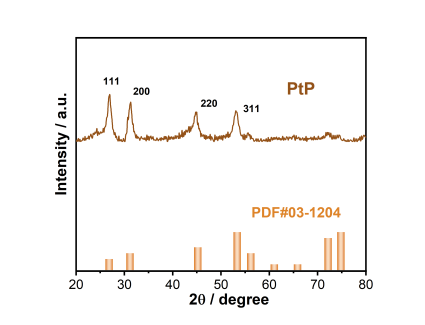

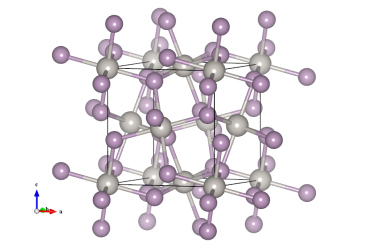

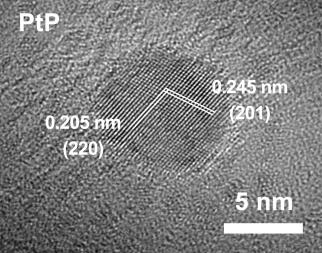

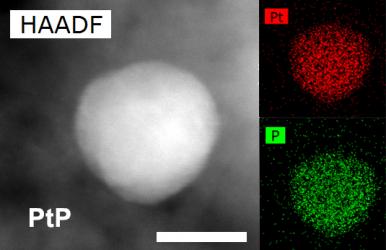

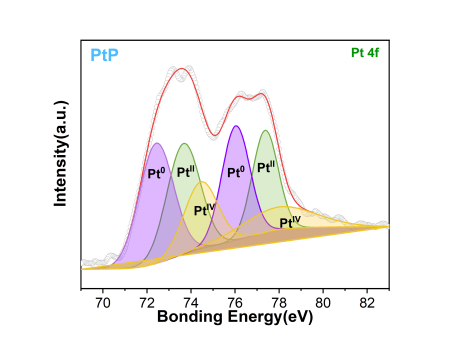


**(d)**

**(f)**

**(e)**

**Figure S15.** (a) The structure of PtP compounds. (b) The XRD patterns of PtP compounds. (c) TEM images of PtP compounds. (d) HRTEM images of PtP compounds. (e) EDS mapping of PtP compounds. (f) XPS of PtP compounds.

**S16. Characterization for PtSe samples**

**(c)**

**(b)**

**(a)**


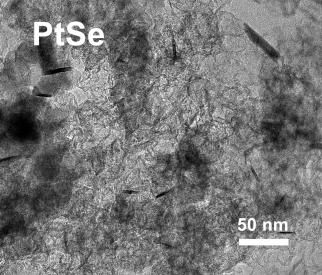

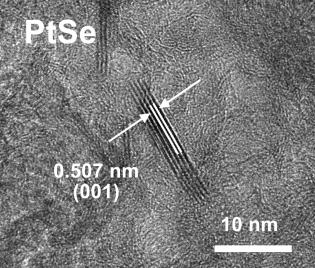

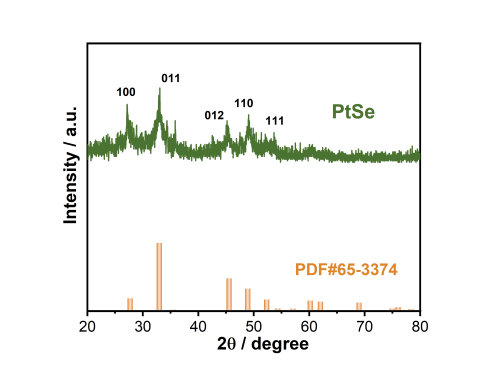

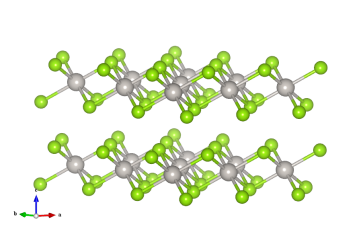

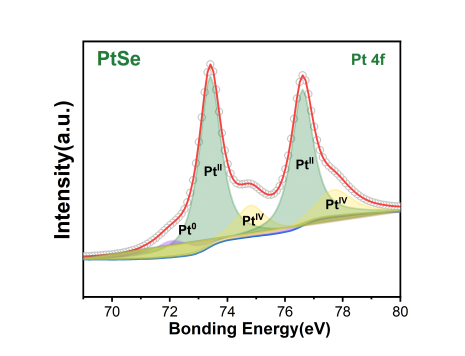

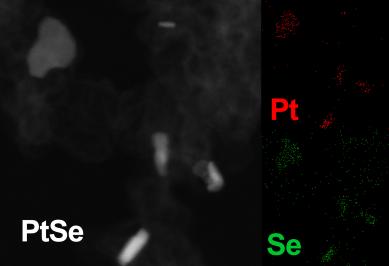


**(f)**

**(e)**

**(d)**

**Figure S16.** (a) The structure of PtSe IMCs. (b) The XRD patterns of PtSe IMCs. (c) TEM images of PtSe IMCs. (d) HRTEM images of PtSe IMCs. (e) EDS mapping of PtSe IMCs. (f) XPS of PtSe IMCs.

**S17. Ultraviolet Photo-electron Spectroscopy analysis**


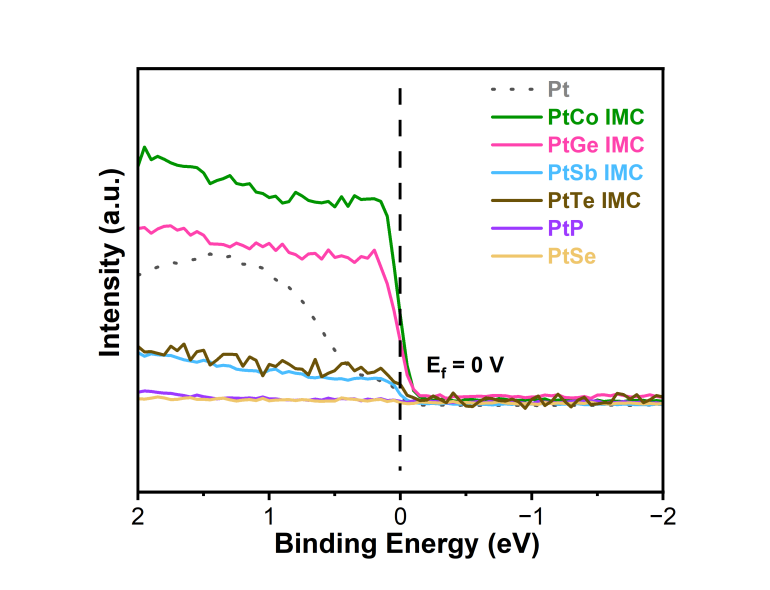

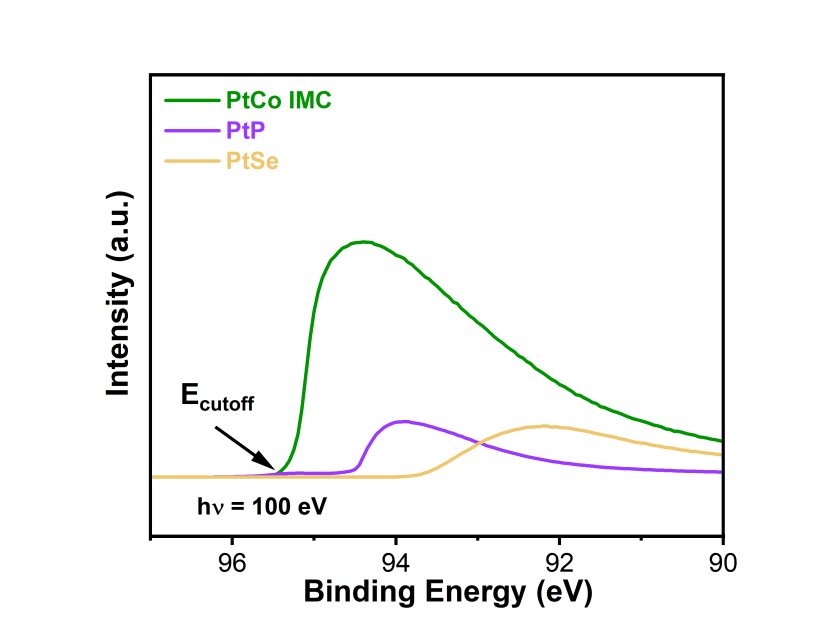


W_f_=hν- (E_cutoff_ - E_f_) ( hν = 100 eV)

**Figure S17.** The UPS spectra of PtCo IMC, PtP and PtSe.

**S18. The Near-edge XAFS results**


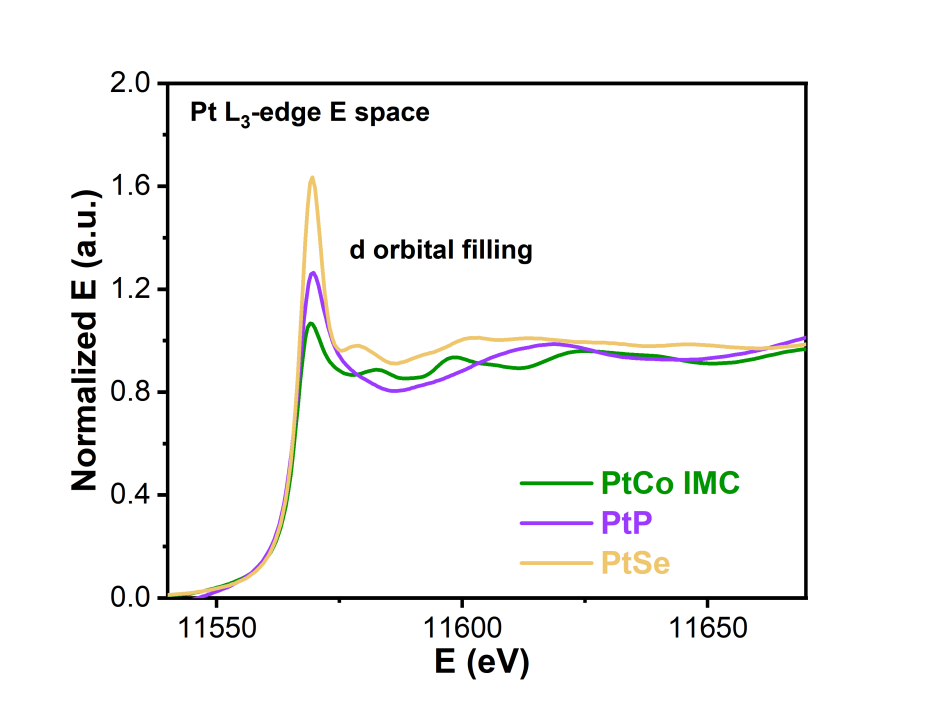


**Figure S18.** The Near-edge XAFS results for as-synthesized PtCo IMC, PtP and PtSe.

**S19. Pt L_3_-edge WT-EXAFS contour plots of semimetal-Pt intermetallics**

**(b)**

**(c)**

**(a)**


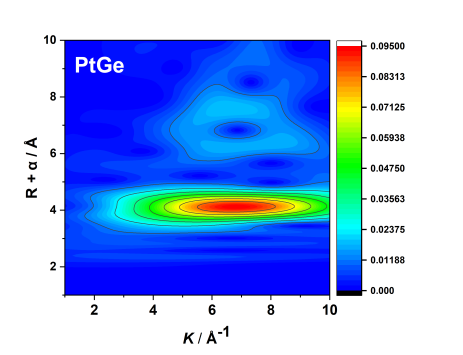

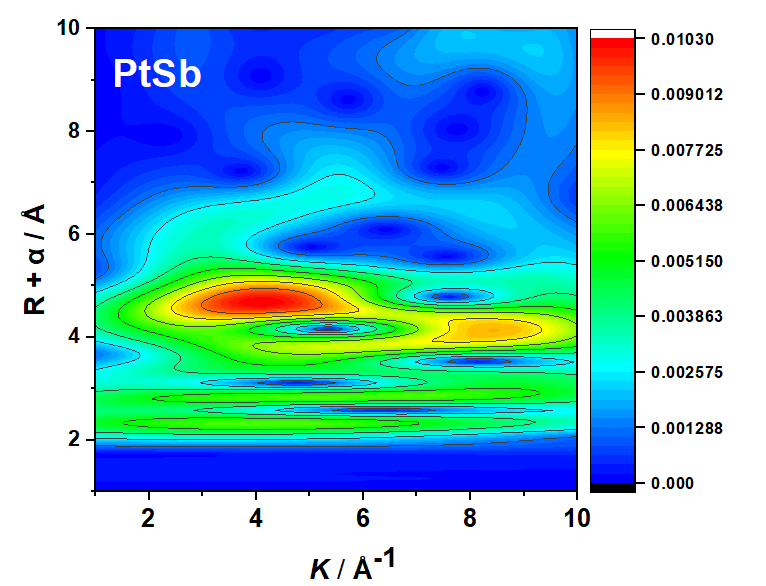

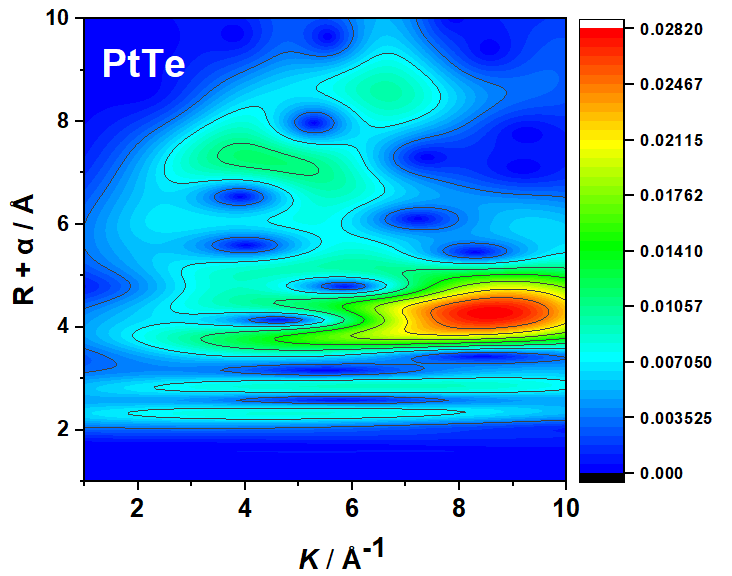


**Figure S19.** The Pt L_3_-edge WT-EXAFS contour plots of semimetal-Pt intermetallics. (a) for PtGe IMC. (b) for PtSb IMC. (c) for PtTe IMC.

**Tale S1. The synthesis temperature for intermetallics**


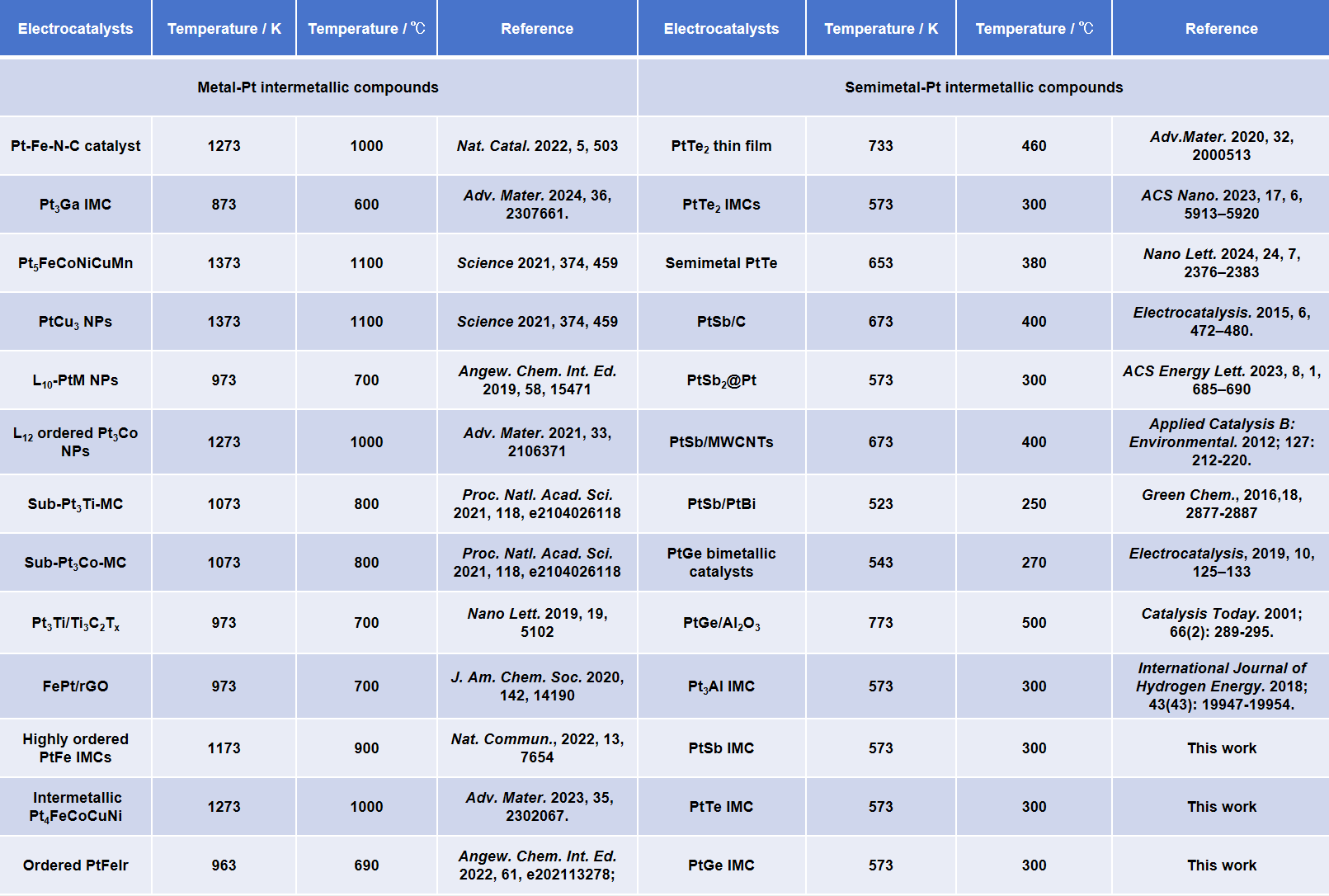


**Table S1.** The comparison of synthesis temperature for Pt-based intermetallic compounds in recent literature.

**Table S2.** **The ICP results for semimetal-Pt IMCs**

| **Content / Pt** | **PtGe IMC** | **PtSb IMC** | **PtTe IMC** |
| --- | --- | --- | --- |
| **Mass ratio** | **23.4 %** | **19.3 %** | **21.8 %** |
| **Atomic ratio** | **2.6 %** | **1.9 %** | **2.2 %** |

**Table S2.** The ICP results for semimetal-Pt IMCs

**Tale S3. The comparison of mass activity and poison tolerance**


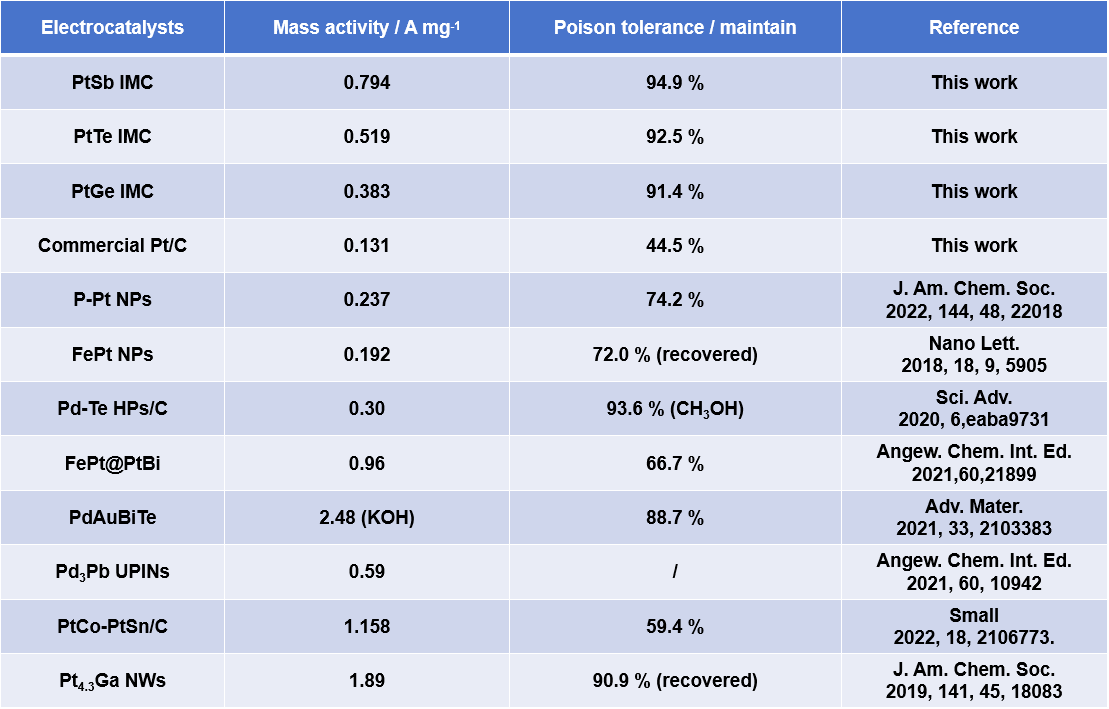


**Table S3.** The comparison of mass activity and poison tolerance for recent reported electrocatalysts.
